# Visualization of weak interactions between quantum dot and graphene in hybrid materials

**DOI:** 10.1038/s41598-017-00542-9

**Published:** 2017-03-24

**Authors:** Shuo Cao, Jingang Wang, Yong Ding, Mengtao Sun, Fengcai Ma

**Affiliations:** 1 Department of Physics and Department of Chemistry, Liaoning University, Shenyang, 110036 P.R. China; 2 Beijing Key Laboratory for Magneto-Photoelectrical Composite and Interface Science, School of Mathematics and Physics, University of Science and Technology Beijing, Beijing, 100083 P.R. China; 3 Beijing National Laboratory for Condensed Matter Physics, Institute of Physics, Chinese Academy of Science, Beijing, 100190 P.R. China; 4 Department of Physics, Shenyang Aerospace University, 110036 Shenyang, P.R. China

## Abstract

The mechanisms of the weak interactions within hybrid materials such as quantum dot (QD) and graphene (GR) have important implications for the design of related optoelectronic devices. We characterize the weak interactions in hybrid QD-GR systems using a non-covalent interactions approach. For a single Cd_13_Se_13_ QD with a core-cage structure, the intensity of the steric repulsive strain in every Cd-Se spatial four-atom ring of the cage surface is stronger than that of the inter-core-cage structure. Van der Waals (vdW) interactions occur within the cavity of the cage and within the six-atom rings of the cage surface. The spatial repulsion strain and attractive interactions play a key role in stabilizing the structure of the monolayer graphene. Interestingly, the spatial six-atom ring of the single QD change into spatial four-atom rings of the QD in the hybrid system, accompanied by the translation of vdW interactions into steric repulsive interactions. We conclude that the vdW interactions with *π* extensions and the weak attractive interactions within local areas between the QD and graphene together stabilize the integral structure of the hybrid QD-GR system. These results explain of the formation mechanism and the stabilization of the components in QD-GR hybrid materials.

## Introduction

Over the past several years, low-dimensional colloidal quantum dots (QDs) and graphene hybrid materials have aroused strong research interest because of their potential applications in novel optoelectronic devices; this potential arises from their unique optical and electronic properties^1–4^. Colloidal QDs exhibit broad absorption spectra. Because they are zero-dimensional nanomaterials with obvious quantum confinement effect, their emission spectra can be easily tuned from the visible-light to the infrared-light region via the low-cost synthesis of QDs of different sizes^5, 6^. QD nanoclusters have been intensively investigated as promising materials for light-emitting diodes for full-colour displays^7, 8^ and light-harvesting materials for solar cells^9, 10^ and fluorescent sensors^11^. By contrast, single-layer graphene, as a two-dimensional hexagonal honeycomb material, possesses high-speed charge carriers and exhibits low light absorption^12, 13^. Because of its extraordinary optical and electric properties, graphene is an excellent candidate for transparent electrodes^14^. Therefore, graphene offers great prospects for complementing QDs in hybrid materials systems, and the mechanisms of energy and charge transfer from QDs to graphene in hybrid QD-GR structures have been intensively explored both experimentally and theoretically^1, 2, 15–18^. In hybrid QD-GR structures, graphene sheets are generally the substrate and QDs lie on them because of weak interactions with graphene^19, 20^. The weak interactions are necessary for forming stable structures including single QD, single-layer graphene and QD-GR hybrid materials; however, the characterization and correlation of low-dimension materials’ different weak interactions, which play a key role in stabilizing the structures, are not clear. In addition, understanding the compatible mechanisms of weak interactions within the hybrid QD-GR structures will be advantageous for designing and fabricating high-performance low-dimensional, integrated optoelectronic devices by combining QDs with graphene.

We can utilize a recently developed analysis method pertaining to non-covalent interaction (NCI)^21^ to observe and understand the different weak interactions within the hybrid QD-GR structures, including attractive interactions, vdW interactions and steric repulsive interactions. The NCI approach can be used to thoroughly analyse the peculiarity of different weak interactions of various inter- and intra-molecular and solid structures. The NCI theory is based on the dependence of the reduced density gradient RDG(r) on the electron density ρ(r)^22^,

1 $${\rm{RDG}}({\bf{r}})=\frac{1}{2{(3{\pi }^{2})}^{1/3}}\frac{|\nabla \rho ({\bf{r}})|}{\rho {({\bf{r}})}^{4/3}}$$

Furthermore, λ_2_, which is the eigenvalue of the electron density Hessian matrix^23^, is another important parameter in the NCI method. The NCI analysis method depends on the values of RDG(r) and ρ(r) from the regions of weak interactions in the 2D scatter graph, in which *ρ*(**r**) is the horizontal ordinate and RDG(**r**) is the vertical coordinate with relatively low numerical values, and the scatter distribution of RDG(**r**) appears similar to tail feathers oriented towards the horizontal axis. Meanwhile, according to the function values of *ρ*(**r**), the sign of *λ*_2_ changes, so the quantity sign(*λ*_2_)*ρ*(**r**) serves as the new horizontal ordinate replacing *ρ*(**r**). The attractive interactions are based on negative values of sign(*λ*_2_)*ρ*(**r**), and the repulsive interactions are based on positive values of sign(*λ*_2_)*ρ*(**r**), all with relatively large *ρ*(**r**) values. The weak interactions actions occur as vdW interactions when the values of sign(*λ*_2_)*ρ*(**r**) approach zero, irrespective of the sign of *λ*_2_^21–25^. Under these circumstances, the 2D scatter graphs can be transformed into 3D colour graphs of RDG isosurfaces based a on colour scale representation of the numerical value range of the sign(*λ*_2_)*ρ*(**r**) values, enabling the weak interactions in the solid structure to be easily observed in 3D space. For the 3D colour graph of the RDG isosurfaces, the deeper blue colours indicate stronger attractive interactions, which correspond to the smaller negative numbers of the horizontal ordinate in the 2D scatter graph, and the deeper red colours indicate stronger repulsive interactions, which correspond to the larger positive numbers of the horizontal ordinate. The green colours indicate the vdW interactions, which correspond to numerical values close to zero regardless of the negative or positive horizontal axis direction. The colours between blue and green indicate gradually weaker attractive interactions, and the colours between green and red indicate gradually stronger repulsive interactions.

In this work, using the NCI approach, we systematically analysed the features of the weak interactions of single QD, single-layer graphene, and hybrid QD-GR structures on the basis of density functional theory (DFT) calculations. By visually observing various weak interactions, we elucidated their rule for maintaining stable structures of the single QD and single-layer graphene; we also observed a point where the characteristics of the weak interactions of the QD in the optimized hybrid QD-GR system differed from those of the single optimized QD. This difference was caused by the slight rearrangement in the structures of the QD with the fully relaxed geometric structures of the hybrid QD-GR system. However, a comparison of the single-layer graphene with the graphene of the hybrid QD-GR system revealed that the weak interactions were mainly the same. Furthermore, we obtained reliable conclusions about the *π*-extension vdW interactions and local-area weak attractive interactions that together contribute the key effects for stabilizing the integrated structures of the hybrid QD-GR materials.

## Results

### Structure model of the quantum dot, graphene and hybrid materials

**Figure 1:**
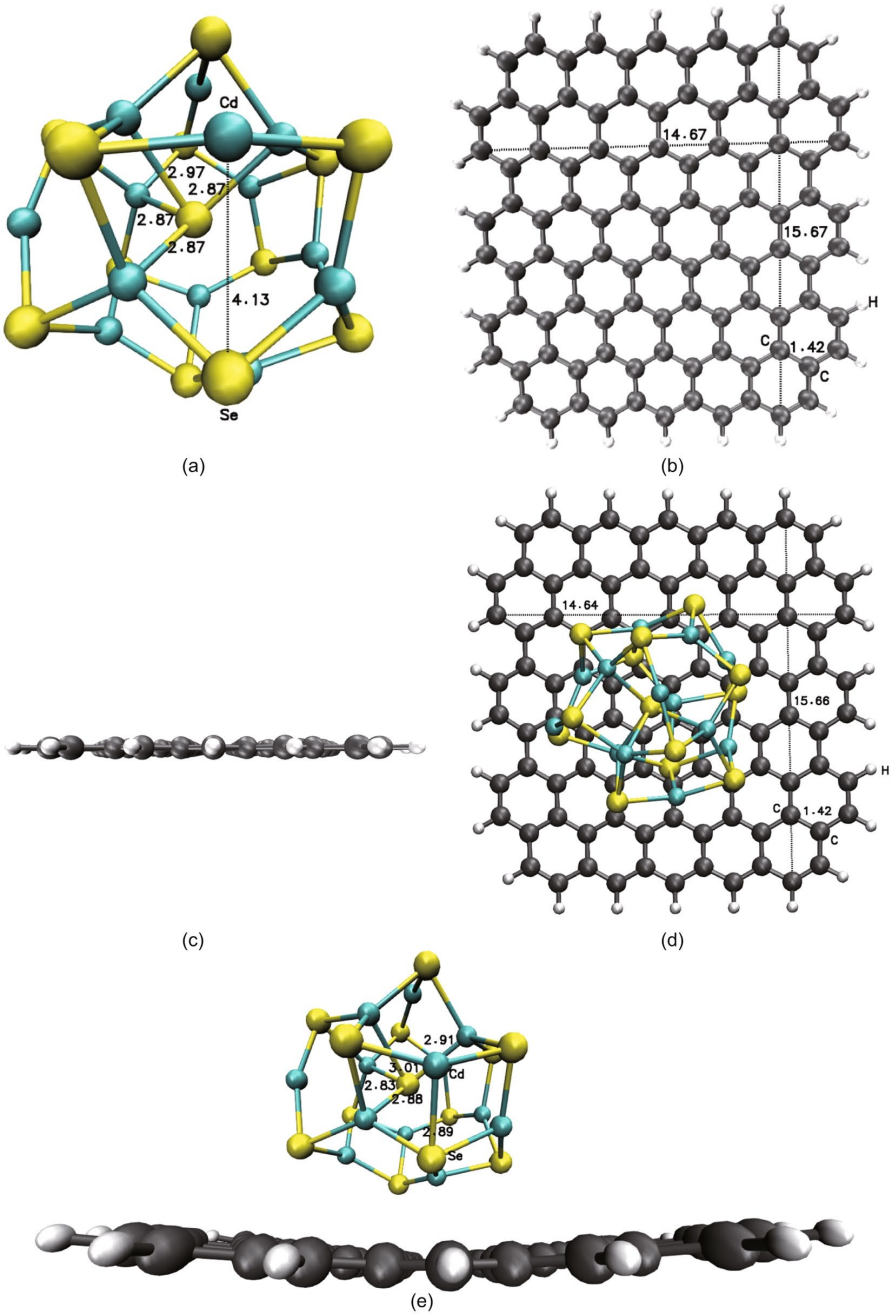
The optimized structure model. (**a**) Single Cd_13_Se_13_ QD with a core-cage structure. The bond lengths between the central Se atoms and four nearby Cd atoms of QD cage surface are 2.87 Å 2.87 Å, 2.87 Å, and 2.89 Å, and the interval between the specified Cd and Se atoms is 4.13 Å. (**b**,**c**) Quadrilateral graphene with two armchair edges and two zigzag edges with top and side views, respectively. The length and width of the single-layer graphene are 15.67 Å and 14.67 Å, respectively, and the lattice constant is 1.42 Å. (**d**,**e**) Hybrid QD-GR structure with top and side views, respectively. The bond lengths between the Se atoms of the QD core centre and four nearby Cd atoms of the QD cage surface in the optimized hybrid QD-GR system become 2.83 Å, 2.88 Å and 2.91 Å and 3.01 Å. The length and width of the quadrilateral graphene with a slightly bent and twistd surface in the optimized hybrid QD-GR system are 15.66 Å and 14.64 Å, respectively.

In this work, the structure model of the optimized single QD as Cd_13_Se_13_ nanostructure is shown in Fig. 1(a); the QD is stable core-cage structure, as confirmed proved by mass spectrometry^26^. Notably, the bond lengths between the central Se atoms and the four nearby Cd atoms of the QD cage surface are 2.87 Å 2.87 Å, 2.87 Å, and 2.89 Å, and the interval between the specified Cd and Se atoms is 4.13 Å, as marked on one of the Cd-Se spatial six-atom rings of the cage surface in Fig. 1(a). The electronic structure of Cd_13_Se_13_ QD has been extensively studied using first-principle methods^27–29^; however, we seek to further elucidate the characterization of weak interactions in the core-cage structure of QD.

The structure model of the optimized single-layer graphene is quadrilateral, as shown in Fig. 1(b,c) with top and side views; this structure includes two armchair edges and two zigzag edges with H atoms linked to the edge C atoms. A similar hexagonal honeycomb structure for graphene was introduced in our previous work^30, 31^. The length and width of the quadrilateral graphene are 15.67 Å and 14.67 Å respectively, and the lattice constant is 1.42 Å, as shown in Fig. 1(b). Experiments and theoretical calculations have demonstrated the electronic structures of graphene with zigzag and armchair edges^32–34^, and we have also further observed the features of the weak interactions within the graphene structure.

Utilizing the aforementioned structural model for the optimized QD and graphene, we combined QD with graphene to form a hybrid QD-GR structure by placing QD in the upper of quadrilateral graphene nanosheet using a DFT geometric relaxation computation, as shown in Fig. 1(d,e) with top and side views. By comparing the structure of the optimized single QD with the structure of the QD in the optimized hybrid QD-GR system, we observed a slight rearrangement of the QD structure in the hybrid system; we observed a similar result for graphene. The bond lengths between the central Se atoms of the QD core centre and four nearby Cd atoms of the QD cage surface of the hybrid QD-GR structure become 2.83 Å, 2.88 Å and 2.91 Å and 3.01 Å (cf. those of the single QDs). Notably, one of the Cd-Se spatial six-atom rings of cage surface in the single QD changes into a double Cd-Se spatial four-atom rings in the hybrid QD-GR structure; the length of the new bond between the specified Cd and Se atoms is 2.89 Å, which is marked among the double Cd-Se spatial four-atom rings of the cage surface in Fig. 1(e), in contrast to the single QD in Fig. 1(a). The geometric structure of graphene in the hybrid system appears to have a slightly bent and twisted surface as compared to the very flat structure of the single-layer grapheme (Fig. 1(d) and (e)), similar to the phenomenon of a small ball placed on and bending a flat sponge. The distance between the bottom of the QD and the top of the graphene surface is approximately 2.5 Å.

### Characterization of the weak interactions in single Cd_13_Se_13_ QD

**Figure 2:**
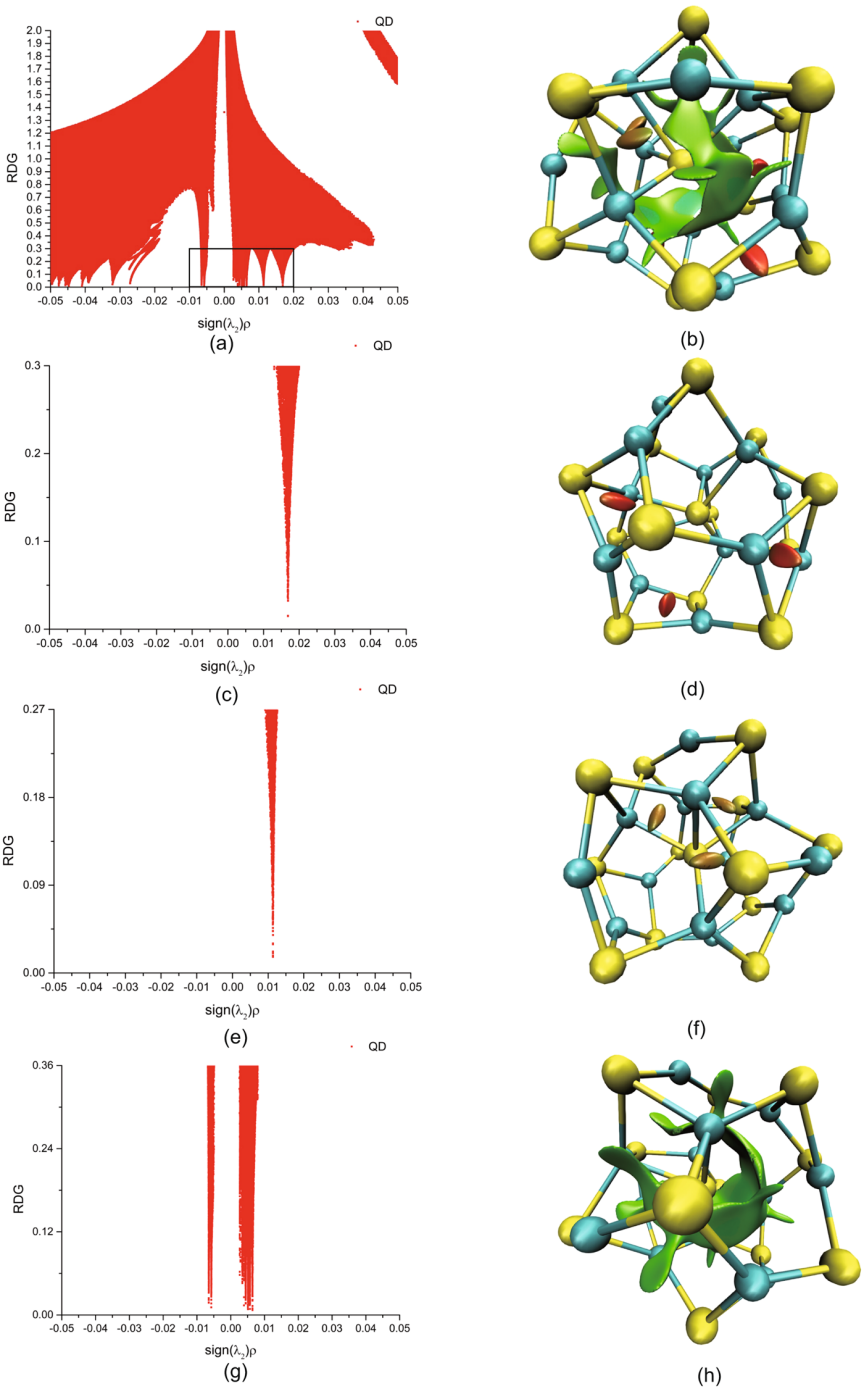
The 2D scatter graphs of single QD and 3D colour graphs of RDG isosurfaces with a blue-green-red colour scale date range corresponding to the values of sign(*λ*_2_)*ρ*(**r**) on the horizontal ordinate of the 2D scatter graph from −0.02 a.u. to 0.02 a.u. (**a**) The 2D scatter graph corresponding to the values of sign(*λ*_2_)*ρ*(**r**) on the horizontal ordinate for the weak interactions from −0.01 a.u. to 0.02 a.u. (**b**) The 3D colour graph of the RDG isosurfaces with a numerical cutoff value of 0.3 a.u. (**c**,**d**) The 2D scatter graph corresponding to the values of sign(*λ*_2_)*ρ*(**r**) for the weak interactions between 0.013 a.u. and 0.02 a.u. and the 3D colour graph of the RDG isosurfaces with a cutoff value of 0.3 a.u., respectively. (**e**,**f**) The 2D scatter graph corresponding to the values of sign(*λ*_2_)*ρ*(**r**) for the weak interactions between 0.008 a.u. and 0.013 a.u. and the 3D colour graph of the RDG isosurfaces with a cutoff value of 0.27 a.u., respectively. (**g**,**h**) The 2D scatter graph corresponding to the values of sign(*λ*_2_)*ρ*(**r**) for the weak interactions between −0.01 a.u. and 0.008 a.u. and the 3D colour graph of the RDG isosurfaces with a cutoff value of 0.36 a.u., respectively.

The 2D scatter graph of single QD is illustrated in Fig. 2(a). To explore the weak interactions, we only focus on low numerical values of RDG, with a cutoff value of 0.3 a.u., and low values of the horizontal axis, sign(*λ*_2_)*ρ*(**r**), with an interval between −0.01 a.u. and 0.02 a.u. in the 2D scatter graph, corresponding to the RDG distribution, which resembles a tail feather structure towards the horizontal axis within the black frame in Fig. 2(a). 3D colour graph of the RDG isosurfaces is shown in Fig. 2(b). Clear characterization of the weak interactions in different parts of the QD, requires analysis of the 2D scatter graphs in detail; we therefore decomposed the horizontal ordinate into three subsections in the 2D scatter graph, as follows:

First, we only kept the values of the RDG that correspond to the interval of numerical values of between 0.013 a.u. and 0.02 a.u. on horizontal ordinate, as shown in Fig. 2(c), and demonstrated the colour graph of the RDG in 3D space with a cutoff value for the isosurfaces of 0.3 a.u., as shown in Fig. 2(d). Three red rice grain-shaped isosurfaces are observed in three similar Cd-Se spatial four-atom rings of the cage surface of the QD, which reflect the steric repulsive strain induced by the rings. Second, we similarly kept the values of the RDG that correspond to the numerical values between 0.008 a.u. and 0.013 a.u. on horizontal ordinate, and the isosurfaces with a cutoff value of 0.27 a.u. Their 2D scatter graph and 3D colour graph are shown in Fig. 2(e) and (f), respectively. Another three olive grain-shaped isosurfaces exist in three Se-Cd spatial four-atom rings of the inter-core-cage of the QD, which indicate the weaker steric repulsive strain induced by the rings inside the cage. Third, we selected the values of the RDG that correspond to the numerical values between −0.01 a.u. and 0.008 a.u. on horizontal ordinate, and the isosurfaces with a cutoff value of 0.36 a.u.; the 2D scatter graph and 3D colour graph are shown in Fig. 2(g) and (h), respectively. The vdW interactions, shown in green, partially encircle the centre Se atoms of the core-cage structure of the QD. Furthermore, the local weaker attractive interactions are shown in cyan between the Se atoms of the cage centre and nearby Cd atoms of the cage surface. However, green elongated board- or pillar-shaped isosurfaces exist in every similar Cd-Se spatial six-atom ring of the cage surface of the QD, which reflects the vdW strain induced by the large rings. The strength of the weak interactions in the QD core-cage structure is clearly discriminative.

### Peculiarity of the weak interactions in the single-layer graphene

**Figure 3:**
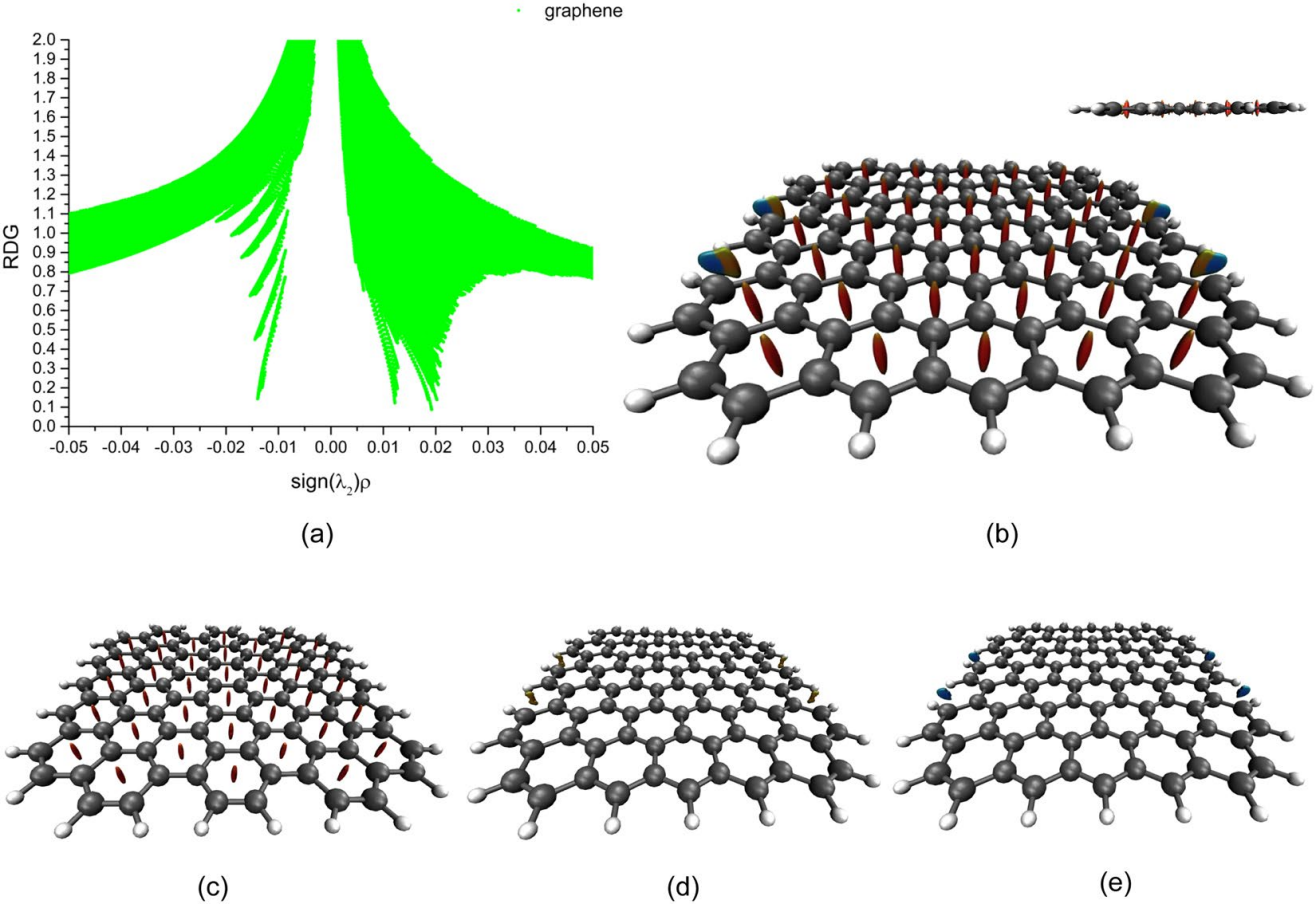
The 2D scatter graphs of single-layer graphene and 3D colour graphs of RDG isosurfaces with blue-green-red colour scale date ranging from −0.02 a.u. to 0.02 a.u. (**a**,**b**) The 2D scatter graph corresponding to the values of sign(*λ*_2_)*ρ*(**r**) on the horizontal ordinate for the weak interactions from −0.02 a.u. to 0.024 a.u. and the 3D colour graph of the RDG isosurfaces with cutoff value of 0.6 a.u., respectively, (the inset shows the side view). (**c**) The 3D colour graph of the RDG isosurfaces with a cutoff value 0.6 a.u. corresponding to the values of sign(*λ*_2_)*ρ*(**r**) for the weak interactions in the 2D scatter graph between 0.014 a.u. and 0.024 a.u. (**d**) The 3D colour graph of the RDG isosurface with cutoff value of 0.5 a.u. corresponding to the values of sign(*λ*_2_)*ρ*(**r**) on the horizontal ordinate for the weak interactions of the 2D scatter graph between 0 a.u. and 0.013 a.u. (**e**) The 3D colour graph of the RDG isosurface with a cutoff value of 0.6 a.u. corresponding to the values of sign(*λ*_2_)*ρ*(**r**) on the horizontal ordinate for the weak interactions of the 2D scatter graph between −0.02 a.u. and 0 a.u.

We used the same method to demonstrate the 2D scatter graph of single-layer graphene in Fig. 3(a) and to show the visualization analysis of the 3D RDG isosurfaces in Fig. 3(b) with a cutoff value of 0.6 a.u., corresponding to the values on the horizontal ordinate between the interval from −0.02 a.u. to 0.024 a.u. in the 2D scatter graph. We analysed the peculiarity of the weak interactions in every section. The 3D colour graph of the RDG isosurfaces that correspond to interval from 0.014 a.u. to 0.024 a.u. on the horizontal ordinate of the 2D scatter graph is shown in Fig. 3(c) with a cutoff value of 0.6 a.u.; we observe red isosurfaces with an extended rugby shape at the centre of every hexagonal ring of six C atoms. The 3D colour graph of the RDG isosurfaces that correspond to the interval 0 a.u. to 0.013 a.u. on the horizontal ordinate of the 2D scatter graph is shown in Fig. 3(d) with a cutoff value of 0.5 a.u.; in this case, we observe dark orange, small, irregular shapes attached to the semi-ring centres of the graphene armchair edges. These observations reveal weak steric repulsive interactions. The 3D colour graph of the RDG isosurfaces corresponding to the interval from −0.02 a.u. to 0 a.u. on the horizontal ordinate is shown in Fig. 3(e) with a cutoff value of 0.6 a.u., and we find blue semi-elliptical shapes among two H atoms of the armchair edges of graphene. These observations reveal strong attractive interactions between two H atoms.

### Features of the weak interactions in the hybrid QD-GR structure

**Figure 4:**
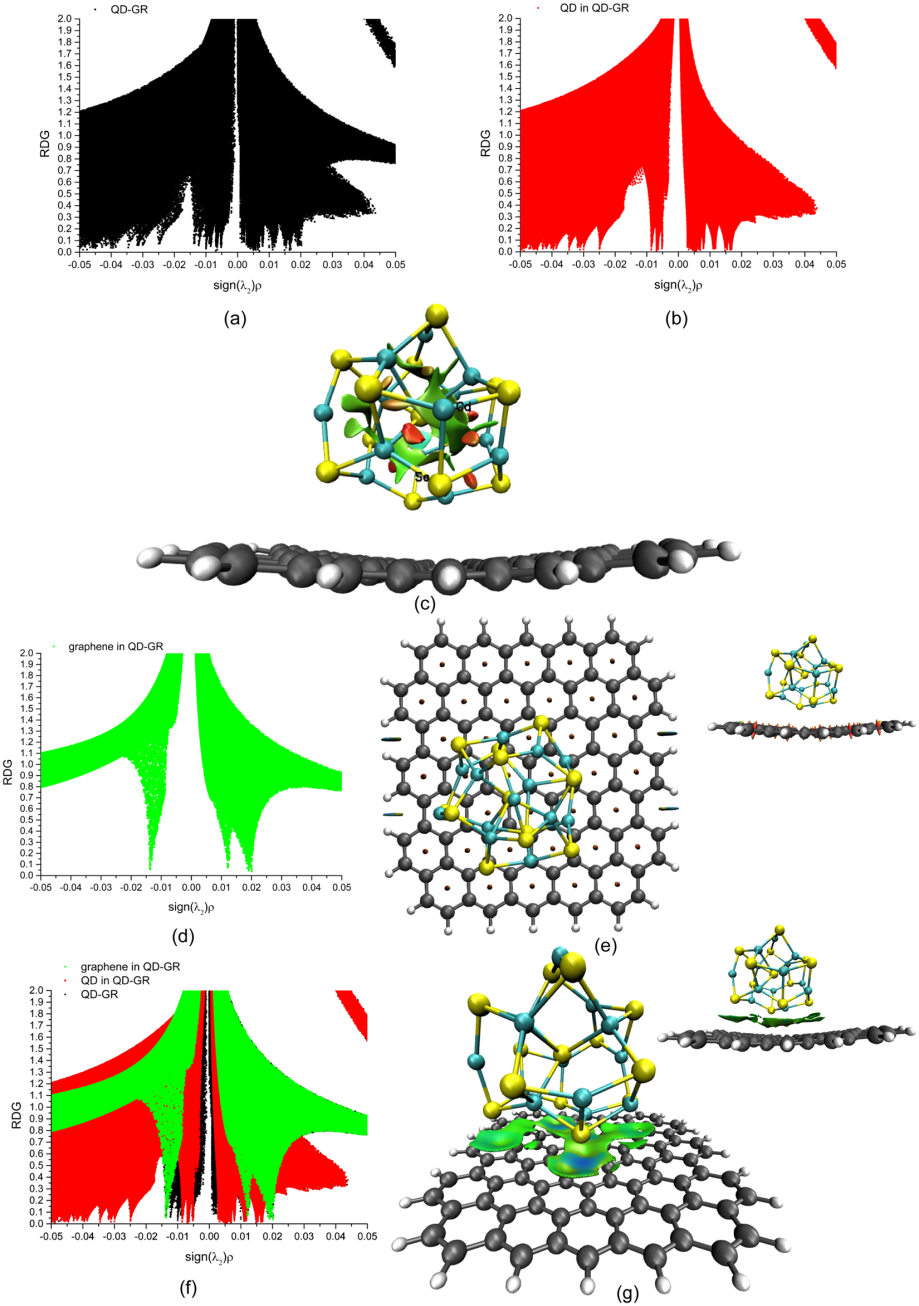
The 2D scatter graphs of the hybrid QD-GR system and the 3D colour graphs of the RDG isosurfaces corresponding to a blue-green-red colour scale date range from −0.02 a.u. −0.02 a.u. (**a**) The 2D scatter graph of the hybrid QD-GR structure. (**b**,**c**) The 2D scatter graph of the QD in the hybrid QD-GR structure and the 3D colour graph of the RDG isosurface with a cutoff value of 0.29 a.u., respectively. (**d**,**e**) The 2D scatter graph of the graphene in the hybrid QD-GR structure and the 3D colour graph of the RDG isosurface with a cutoff value of 0.6 a.u., respectively, (the inset shows the side view). (**f**) The 2D overlap scatter graph of the QD, the graphene and hybrid structure in the hybrid QD-GR system. (**g**) The 3D colour graph of the RDG isosurface between the QD and graphene in hybrid QD-GR structure with a cutoff value of 0.6 a.u., (the inset shows the side view).

The 2D scatter graph of the hybrid QD-GR structure is shown in Fig. 4(a), and the RDG distributions for the region of weak interactions are relatively complicated. To easily distinguish the roles of the weak interactions in the main region of the 2D scatter graph of the hybrid QD-GR structure, we unambiguously demonstrated the corresponding 3D colour graph of RDG isosurfaces. We translated the corresponding entire cube grid surrounding the whole QD-GR structure into two-part cube grid, and made the cube grid to surround the QD and graphene in the hybrid QD-GR system.

We then plotted the 2D scatter graph of the QD in the hybrid QD-GR structure shown in Fig. 4(b). The corresponding 3D colour graph of the RDG isosurfaces with a cutoff value of 0.29 a.u. is shown in Fig. 4(c). This graph exhibits similar colours and shapes for isosurfaces compared to the graph of the single QD in Fig. 2(b), except for a few obvious differences. By comparing the geometric structure of the QD in the hybrid QD-GR system with single QD, we found that the geometric structure had slightly changed, but that the core-cage structures of the QDs were similar. One obvious difference was observed in that the new emerging double spatial four-atom rings of the QD in the hybrid system, shown in Fig. 1(e), replaced one of the spatial six-atom rings of the single QD, as shown in Fig. 1(a). On the basis of the NCI analysis, we found two red rice grain-shaped isosurfaces with steric repulsive interactions in the double spatial four-atom rings of the QD in the hybrid system, as shown in Fig. 4(c); these interactions are distinctly different from the vdW interactions of the spatial six-atom rings of the single QD, as shown in Fig. 2(h).

The 2D scatter graph of the graphene in the hybrid QD-GR system is shown in Fig. 4(d), and the 3D colour graph of the RDG isosurfaces with a cutoff value of 0.6 a.u. is presented in Fig. 4(e). By comparing Figs 3(a) and 4(d), we observed the phenomenon where the two 2D scatter graphs were approximately the same, and the 3D colour graphs were similar when comparing Figs 3(b) and 4(e), even if the spatial structure of the graphene in the hybrid QD-GR system was bent slightly more than the two-dimensional flat single-layer graphene.

To distinctly analyse the features of the 2D scatter graph of the hybrid QD-GR structure, we plotted the 2D scatter graph of the QD and graphene in the hybrid QD-GR system and that of the whole hybrid QD-GR structure in an overlap graph, as shown in Fig. 4(f). By excluding the contribution from the 2D scatter graph of the QD and graphene in the QD-GR system, we observe that the new weak interactions increased and that the new tail feathers distribution of the low numerical values of the RDG that point to the horizontal axis appeard. Most importantly, on the basis of the aforementioned analysis of the 2D scatter graph and 3D colour graph of the RDG isosurfaces of the QD and graphene in the hybrid QD-GR system, we show exclusively in Fig. 4(g) the corresponding 3D colour graph of the RDG isosurfaces between the QD and graphene by shielding the 3D colour graph of the inter-QD and inter-graphene interactions in the hybrid QD-GR system. However, we clearly observe green large areas in the 3D colour graph of the RDG isosurfaces between the QD and graphene in the QD-GR hybrid structure in Fig. 4(g) with a cutoff value of 0.6a.u., and elucidate that attractive interactions also appeared between the Se atoms of the lower part of the QD and the upper surface of the graphene, these interactions shown in light blue. Therefore, this figure visually exhibits the superposition effect of the weak vdW interactions with a *π* bond distribution and the localized attractive interactions.

## Discussion

Because of the noticeable phenomena observation using the NCI analysis method, we can now discuss the characterization of the weak interactions of the single QD, single-layer graphene, and the hybrid QD-GR structure.

In the case of single QD, by comparing the Cd-Se spatial-four atom rings to the spatial six-atom rings of the cage surface, we generalized that the strength of the weak interactions observably decreased, and that the strength of the weak interactions of the Se-Cd spatial four-atom rings within the cage of the QD was of medium intensity. In addition, the vdW interactions encircled the cavity inside the core-cage of the QD and provided a fundamental support frame for stabilizing the single QD structure. All of the aforementioned weak interactions together played an important role in stabilizing the single QD core-cage structure.

Regarding the single-layer graphene, we concluded that the strong steric stabilization due to repulsive strain in all six hexagonal rings of the C atoms stabilized the entire structural framework of the single-layer graphene. After observing the weak interactions of the armchair edges shown in Fig. 3(b), we observed two-coloured dark orange/blue isosurfaces, which revealed the fact that the weak repulsive and strong attractive interactions jointly functioned to stabilize the armchair edge structures of the single-layer graphene.

In the case of the hybrid QD-GR structure, the weak interactions of the QD in the hybrid system had slight difference and exhibited novel characteristics compared to the single QD. The QD in the hybrid system exhibited a slight structural rearrangement compared to the single QD, and observed an interesting phenomenon where altered weak interactions could verify the transformation of the structure. We observed that the spatial six-atom ring of the single QD shown Fig. 1(a), changed into double spatial four-atom rings for the QD in the hybrid system shown in Fig. 1(e); this change was accompanied by the vdW interactions becoming sterically repulsive interactions. Moreover, the intensity of the weak interactions of the QD in the hybrid structure, which is related to the colour and shape of the RDG isosurfaces in the 3D space, exhibited a slight difference in contrast compared to that in the single QD, because the geometric structure partly rearranged after the hybrid QD-GR structure fully relaxed while maintaining the stabilization of the whole the core–cage structure. The weak interactions of the graphene in the hybrid system were similar to those of the single-layer flat graphene. Although slightly twisted graphene structure was observed in the hybrid system, the similar weak interactions could maintain the structural stabilization. However, the vdW interactions within the big *π*-extension areas and the weak attractive interactions within the local areas between the QD and graphene together played a key role in maintaining the stabilization of the integral structure of the hybrid QD-GR system.

In summary, we visually demonstrated the mechanisms of the weak interactions of single QD, single-layer graphene and hybrid QD-GR structure by utilizing the NCI approach and concluded that the attractive interactions, steric repulsive interactions, and vdW interactions cooperate in stabilizing the intrastructure of low-dimensional QD or graphene materials. Generally, in the case of single QD or QD in the hybrid system, similar weak interactions strongly affected the stability of the structure of the core-cage. Unexpectedly, we found that the weak interactions are advantageous for affirming the transformation of the QD structure in the hybrid system, in contrast to the single QD. By comparison with the single-layer graphene, we observed the phenomenon where a slight structural twist of the graphene in the hybrid system did not change the peculiarity of the weak interactions. In this work, we visually demonstrated the overlay effect of weak interactions due to the complex QD-GR structure, the superposition effect of *π*-extended vdW interactions and localized weak attractive interactions between the hybrid QD and the graphene system for stabilizing the integrated structure of the hybrid QD-GR system. This study improves existing knowledge of such complex materials at a fundamental level and promotes a deep understanding of the mechanisms of weak interactions for relevant integrated optoelectronic devices.

## Methods

We optimized the geometric structure of low-dimensional QD and graphene materials via based DFT method^35^ by using the long-range hybrid functional ωB97X-D^36^, which provides superior results with respect to non-bonding interactions. The basis set, LANL08, derived from the EMSL Basis Set Exchange Library^37^ was used for the Cd and Se atoms, and the 6–31 g(d) basis set was applied for the C and H atoms. All of the calculations were conducted using the Gaussian 09 software package^38^. The 2D scatter graph data and the cube grid data of the RDG isosurfaces for the 3D visual analysis of the weak interactions were obtained using the Multiwfn software^39^ on the basis of the approach of visualizing the non-covalent interaction. To study the 3D colour graphs of the RDG isosurfaces of the different structures in all experiments with the same criteria, we used the VMD software^40^ to vary the colour scale date range corresponding to the horizontal ordinate in the 2D graphs from −0.02 a.u. to 0.02 a.u.
